# Intervertebral disc degeneration is accelerated by GH overexpression but attenuated by GH antagonism

**DOI:** 10.1007/s11357-025-02022-9

**Published:** 2025-12-02

**Authors:** Abhijit Sukul, Siqi Ren, Anna E. Miller, Huanhuan Liu, Olanrewaju Akande, Simon Y. Tang, Patrick O’Connor, John J. Kopchick, Shouan Zhu

**Affiliations:** 1 Department of Biomedical Sciences, Heritage College of Osteopathic Medicine (HCOM), Ohio University, Athens, OH 45701 USA; 2 Ohio Musculoskeletal and Neurological Institute (OMNI), Heritage College of Osteopathic Medicine (HCOM), Ohio University, Athens, OH 45701 USA; 3 Diabetes Institute (DI), Heritage College of Osteopathic Medicine (HCOM), Ohio University, Athens, OH 45701 USA; 4 Institute of Molecular Medicine and Aging (IMMA), Ohio University, Athens, OH 45701 USA; 5 Department of Orthopedics, Washington University, St. Louis, MO 63130 USA

**Keywords:** Growth hormone, Intervertebral disc, Degeneration, Antagonism

## Abstract

**Background:**

Intervertebral disc degeneration (IVDD) is a leading contributor to lower back pain, yet its underlying mechanisms remain poorly defined. While insulin-like growth factor 1 (IGF-1) has been implicated in disc homeostasis, the specific contributions of growth hormone (GH)—a key upstream regulator of IGF-1 that also acts and independently influences skeletal tissues—have not been thoroughly investigated in the context of IVDD.

**Methods:**

We utilized transgenic mouse models with altered GH signaling, including bovine GH (bGH)-expressing and GH receptor antagonist (GHA) mice, to investigate how GH excess or GH antagonism influences disc structure, degeneration, and to identify potential molecular pathways involved in disc maintenance and degradation. Disc histology was evaluated using Safranin-O/Fast Green staining and quantitative scoring across the nucleus pulposus (NP), annulus fibrosus (AF), and endplate (EP) compartments. Bulk RNA sequencing was conducted on intervertebral discs harvested from 12-month-old bovine GH-overexpressing (bGH) and wild-type (WT) mice to identify transcriptional changes associated with GH overexpression.

**Results:**

bGH mice developed visible IVDD as early as 3 months of age, with degenerative features across all disc compartments. At 15 months, bGH males exhibited more severe degeneration than females, including greater proteoglycan loss, disc height reduction, and fibrotic remodeling. In contrast, 2-year-old GHA mice were protected from age-related disc degeneration, maintaining disc integrity and low histological scores. Transcriptomic analysis revealed sex-specific upregulation of inflammatory and catabolic pathways in bGH discs, including TNF, IL-17, and NOD-like receptor signaling, alongside downregulation of anabolic and ECM maintenance pathways such as PI3K-Akt and mTOR. Notably, these effects were more pronounced in males than females.

**Conclusion:**

Abundance of GH accelerates IVDD through sex-dependent activation of inflammatory and matrix-degrading pathways, while GH antagonism confers protection against age associated disc degeneration. These findings identify GH as a critical regulator of disc homeostasis and a potential therapeutic target for mitigating IVDD and associated low back pain.

**Graphical Abstract:**

**Figure:**
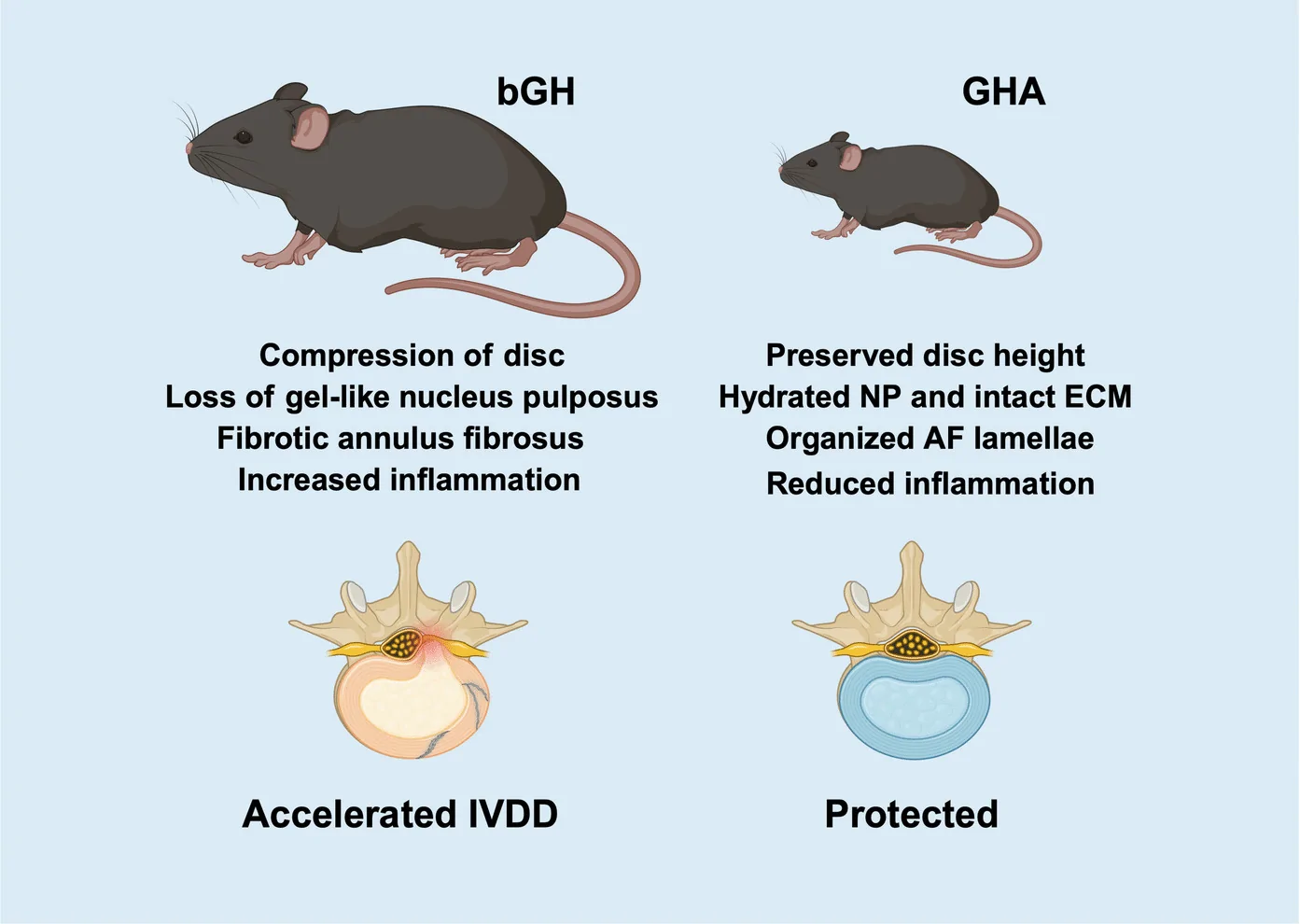

**Supplementary Information:**

The online version contains supplementary material available at https://doi.org/10.1007/s11357-025-02022-9.

## Introduction

Lower back pain (LBP) affects 80% of adults, causing extensive suffering, decreased mobility and significantly burdening socioeconomic status [1–3]. Considerable efforts are made to understand the pathogenesis and optimize the management of LBP [4, 5]. Among many, intervertebral disc degeneration (IVDD) is a substantial cause of LBP; however, underlying mechanisms of IVDD are poorly understood [6–8]. Current treatment strategies primarily focus on symptomatic relief using non-steroidal anti-inflammatory medicines (NSAIDs) and/or surgical intervention [9].

Growth hormone (GH) is responsible for growth in animals and humans by promoting bone growth via chondrocyte proliferation in epiphyseal cartilage at the growth plate [10–12]. Patients with acromegaly, a hormonal disorder in adults that is typically caused by a benign GH-secreting pituitary adenoma, over-produce GH and typically have intervertebral disc degeneration and other issues of the vertebral column, including vertebral body fractures and spinal stenosis [13–16]. These patients often report of LBP due partial-to-complete lumbar disc herniation [16]. Insulin-like growth factor 1 (IGF-1) is regulated by GH and plays a crucial role in promoting cell survival, synthesizing the extracellular matrix (ECM), and maintaining tissue homeostasis, and thus, proper function, within the IVD. It primarily achieves this by stimulating the proliferation of disc cells and enhancing the production of proteoglycans and type II collagen [17–21]. These anabolic effects are crucial for maintaining the hydration and mechanical resilience of the nucleus pulposus, both of which are essential for preserving disc integrity. GH stimulates the liver and other tissues to produce IGF-1, which mediates many of GH’s anabolic and growth-promoting effects. IGF-1, in turn, provides negative feedback to the pituitary and hypothalamus to regulate GH secretion [22, 23]. The actions of GH on IVDD have not been substantially investigated.

Various mouse models with altered GH actions have been utilized to investigate the role of GH on skeletal development and diseases [24–29]. For example, the bovine growth hormone (bGH) overexpression mouse line is often used to study acromegaly [24, 30], a condition characterized by a range of skeletal and ligamentous deformities. These mice show increased joint pathology, including frequent cartilage clefts, thicker synovium, hypertrophic chondrocytes, thinning of the subchondral plate, and generalized joint inflammation [26, 31, 32]. By contrast, antagonizing the GH receptor through a single amino acid substitution (Gly119Arg in bovine GH) leads to the creation of GH antagonist (GHA) mice, in which the mutant hormone acts as a dominant-negative, loss-of-function variant that competitively inhibits endogenous GH signaling [33, 34]. GHA mice are characterized by increased white adipose tissue in several depots and a slight improvement in glucose metabolism [33, 35]. Importantly, we previously also reported that GHA mice are protected from age-associated joint degeneration [31]. However, the consequences on the development of IVDD in mice with differential GH expression remain unknown.

In this study, we utilize multiple mouse models with altered GH signaling—including GH excess and antagonism—to investigate how GH influences intervertebral disc structure, cellular composition, and age-related degeneration. We also performed RNA-Seq to identify GH-regulated molecular pathways that may contribute to IVDD.

## Materials and methods

### Animal models and experimental design

All experiments were conducted according to mouse protocols approved by Ohio University’s Institutional Animal Care and Use Committee (IACUC). WT, bGH [30], and GHA [33, 34] mice were bred and terminally dissected in the Kopchick Laboratory at the Institute of Molecule Medicine and Aging (IMMA) at Ohio University. These mice were kept in ventilated habitats in a temperature-regulated environment, that was maintained at 22 °C, following a 14-h light and 10-h dark cycle, and given ad libitum access to standard rodent chow (ProLab RMH 3000) and water [36]. We assessed male and female WT and bGH mice at 3 and 15 months of age (3 month: WT male *n* = 4, WT female *n* = 7; bGH male *n* = 4, bGH female *n* = 7; 15 month: WT male *n* = 3, WT female *n* = 8; bGH male *n* = 5, bGH female *n* = 9). Additional male WT (*n* = 6) and male GHA (*n* = 5) mice were aged to approximately 2 years to evaluate long-term effects of GH antagonism on disc morphology. Simultaneously, we conducted bulk RNA sequencing on pooled IVD samples collected from 12-month-old WT and bGH in both sexes (each group, *n* = 9) to characterize gene expression profiles.

### Spine tissue processing and analysis

Vertebral column extraction, disc isolation, and tissue preservation protocols followed those outlined in the previous publication [37] and briefly described here. Isolated intervertebral discs (IVD) were fixed in 4% formaldehyde for 48 h at 4 °C and decalcified in formic acid for 72 h at the same temperature, ensuring thorough preparation for histological examination. After decalcification, the samples were dehydrated and embedded intact into the paraffin. Discs were then serially sectioned through the coronal (axial) plane at a thickness of 9 μm. Three to four thin sections were collected on each glass slide for detailed staining and visualization. Two slides were deparaffinized, rehydrated, and sequentially stained with Fast Green and Safranin-O to analyze intervertebral disc degeneration and cartilage integrity. IVDs were evaluated based on disc space, cellular morphology, and matrix integrity for quantifiable measures of degeneration across samples, consisting of the nucleus pulposus (NP), annulus fibrosus (AF), and endplate (EP) according to the histological scoring described in previous literature [38].

### Sample collection and total RNA extraction

IVD samples from 12-month-old WT and bGH mice were collected and pooled by sex and genotype (*n* = 9/group). After collecting, the samples were immediately frozen in liquid nitrogen and transferred to a −80 °C freezer for storage until processed for total RNA extraction. Samples were homogenized using a bead beater in a round-bottom tube containing 500 µL TRIzol reagent (Invitrogen, Carlsbad, CA), and total RNA was extracted following the included protocol. Total RNA quality and concentration were measured with a NanoDrop spectrophotometer. We collected samples from nine biological replicates per group, and to ensure equal representation in the final RNA pool, input volumes were normalized based on RNA concentration prior to pooling. The RNA quality, 600 ng of total RNA, was loaded into a 1% agarose gel containing ethidium bromide and 2% bleach [39]. The total RNA samples were kept at −80 °C and subsequently sent to Novogene for sequencing.

### Library preparation for RNAseq

RNA samples were first assessed using a Bioanalyzer 5400 Expert system (Agilent, Santa Clara, CA) to ensure these optimal characteristics. This device provides precise RNA concentration, integrity, and purity measurements essential for high-quality library construction. Following RNA quality assessment, libraries were prepared using the TRUEseq RNA Library Prep Kit (Illumina, San Diego, CA). This kit employs the purification and fragmentation of RNA, synthesis of first-strand cDNA followed by second-strand cDNA, end repair, A-tailing, adapter ligation, and enhancement PCR. The constructed libraries were sequenced on the NovaSeq X Plus.

### RNAseq data analysis

The Ohio Supercomputer Center (OSC) Owens cluster was used for data collection and processing. Raw sequencing data (fastq format) was downloaded from Novogene data storage. The raw data quality was checked twice before and after trimming using the FASTQC (v0.11.7). After a quality check, the QC-ed data were trimmed using Trimmomatic (v0.36) [40]. After final quality control, data were aligned with the mouse reference genome (GRCm39) for gene analysis using the alignment software Hisat2 (v2.1.0) [41]. After quantifying, the gene read counts were determined using an inner module *featureCount* of subread (v1.5.0) [42]. The read counts for each sample were used for differential expression analysis. Differential gene expression was conducted to identify differentially expressed genes compared to WT control. For differential gene expression with the DESeq2 package [43], additional R packages such as *clusterProfiler*, *biomaRt*, and *Pathview *[44–46] were used to improve data visualization and interpretation, maintaining accuracy and consistency in our statistical evaluation.

### Statistical analysis

To assess histological scores, we performed a student's t-test to determine statistical significance (*P < 0.05, **P < 0.01, and ***P < 0.001). For differential gene expressions in RNAseq analysis, a log fold change threshold above 1 or below −1 and a *p*-value cutoff of 0.05 were considered. To ensure reliability in our results, we calculated the false discovery rate (FDR) using the Benjamini–Hochberg method [47]. All statistical analyses were conducted in R version 4.4.1 or PRISM10.

## Results

### bGH mice develop accelerated IVDD in both sexes

**Fig. 1:**
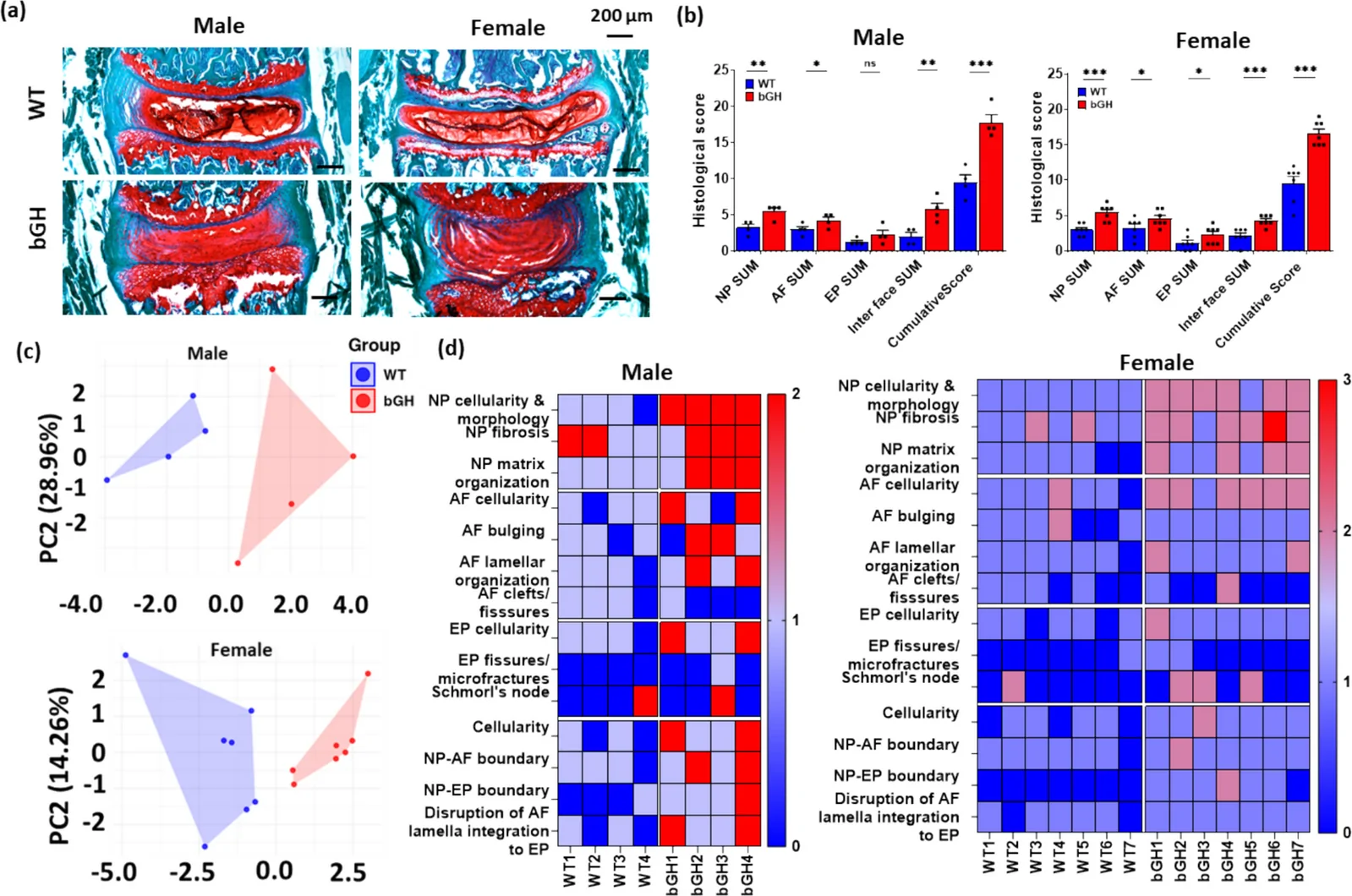
Evaluation of Intervertebral Disc (IVD) Degeneration in 3-Month-Old bGH Mice. (**a**) Representative Safranin O/Fast Green-stained sections of lumbar IVD from WT and bGH mice of both sexes. Top row: WT mice, showing well-preserved disc architecture. Bottom row: bGH mice, exhibiting progressive disc degeneration with abnormal morphology. Scale bar, 200 µm. (**b**) Quantitative scoring analysis: Bar graph summarizing cumulative histopathological scores across IVD regions. (**c**) Principal Component Analysis (PCA) illustrating clear separation between WT and bGH mice based on histopathological features. (**d**) Heatmap Visualization: Heatmap displaying regional histopathological scores for the nucleus pulposus (NP), annulus fibrosus (AF), and cartilaginous endplates (EP), comparing WT and bGH groups. Statistical significance is indicated where applicable; error bars represent mean ± SEM. Significance levels are indicated by asterisks or not significant (ns), *P < 0.05, **P < 0.01, and ***P < 0.001)

At 3 months of age, WT mice intervertebral discs displayed uniform and intact proteoglycan distribution, with no signs of fibrosis or disc space narrowing. However, the bGH mice exhibited early signs of disc degeneration, including reduced proteoglycan content and increased fibrosis, both of which were especially pronounced in the NP area, especially in NP cellularity and matrix organization (Fig. 1a; Figure S4). Additionally, a greater number of hypertrophic chondrocytes were observed in the bGH growth plate compared to WT, as evidenced by enlarged cell morphology and expanded lacunae within the cartilage endplate region (Fig. 1a; Figure S4). To assess potential regional differences in pathology, we plotted the summed histological scores for each IVD compartment, as well as the total cumulative score across all compartments (Fig. 1b). The principal component analysis (PCA) plot summarizes all pathological scores and shows a clear separation between WT and bGH groups (Fig. 1c), consistent with overall higher degeneration scores in bGH. Figure 1d summarizes the histological scoring parameters for individual mice. bGH mice exhibited higher scores across almost all parameters, particularly in NP cellularity, NP fibrosis, NP matrix organization, annulus fibrosus (AF) lamellar organization, end plate (EP) cellularity, Schmorl’s node formation, and the boundaries between NP, AF, and EP. bGH mice exhibited statistically significant increases in scores in each compartment compared to WT controls.

**Fig. 2:**
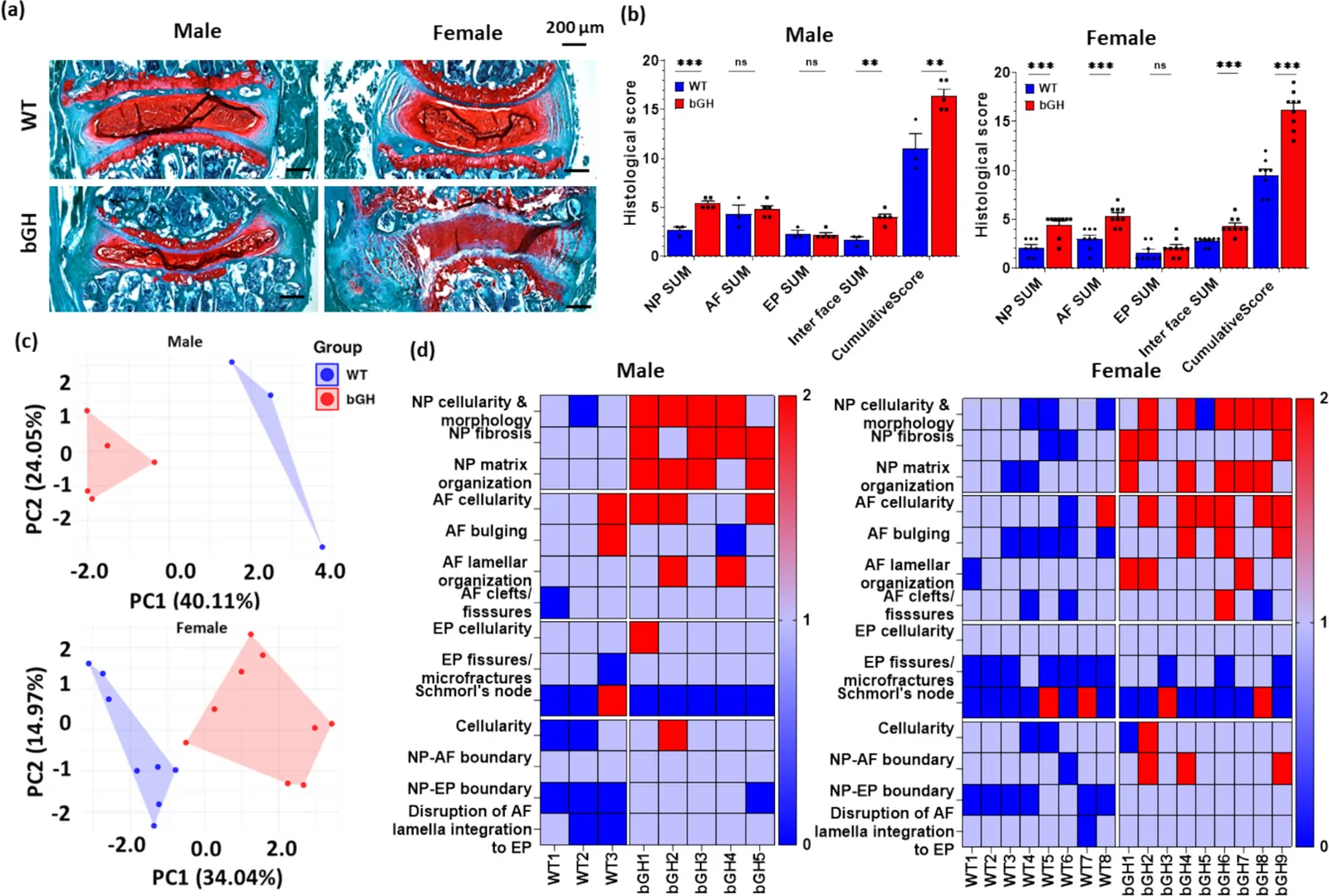
Evaluation of Intervertebral Disc Degeneration in 15-Month-Old bGH Mice. (**a**) Representative Safranin O/Fast Green-stained sections of lumbar IVD from WT and bGH mice of both sexes. Top row: WT mice, showing well-preserved disc architecture. Bottom row: bGH mice, exhibiting progressive disc degeneration with disrupted morphology. Scale bar, 200 µm. (**b**) Quantitative scoring analysis: Bar graph summarizing cumulative histopathological scores across IVD regions. (**c**) Principal Component Analysis (PCA) illustrating clear separation between WT and bGH mice based on histopathological features. (**d**) Heatmap Visualization: Heatmap displaying regional histopathological scores for the nucleus pulposus (NP), annulus fibrosus (AF), and cartilaginous endplates (EP), comparing WT and bGH groups. Statistical significance is indicated where applicable; error bars represent mean ± SEM. Significance levels are indicated by asterisks or not significant (ns), *P < 0.05, **P < 0.01, and ***P < 0.001)

We further analyzed bGH and WT mice at 15 months of age; the markedly shortened lifespan of bGH mice requires that we use 15-month animals to represent the fully senescent life stage [48, 49]. At 15 months of age, both male and female bGH mice exhibited clear signs of disc degeneration; however, the severity was greater in males. Common degenerative features included reduced disc integrity, substantial proteoglycan depletion, and fibrotic remodeling (Fig. 2a; Figure S5). While male bGH mice exhibited significant disc height loss, female bGH mice maintained relatively better disc height. Nonetheless, both sexes had significantly higher cumulative histological scores compared to WT, with male scores indicating more severe degeneration across all disc compartments (Fig. 2b). The PCA plot (Fig. 2c) showed clear separation between WT and bGH mice, indicating distinct histopathological profiles. We further stratified the scores by different parameters, revealing elevated scores across all parameters in both sexes and with males showing more pronounced changes when comparing WT and bGH. In particular, differences were noted in NP cellularity, matrix disorganization, and fibrotic changes. Females also demonstrated increased NP cellularity and AF disorganization, but to a lesser extent than that observed in males (Fig. 2d).

### Antagonism of GH action protected mice from developing IVDD

**Fig. 3:**
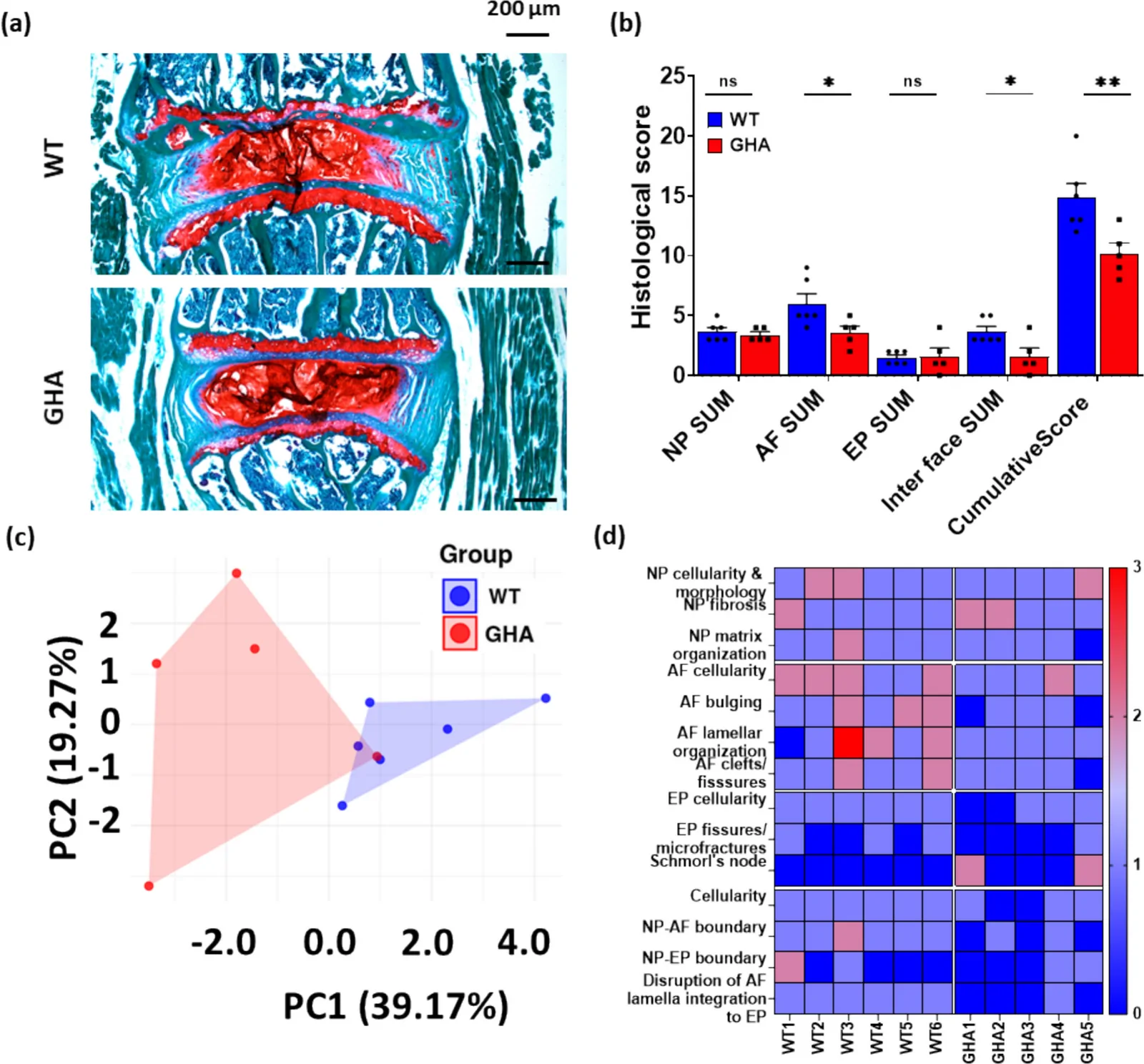
Evaluation of Intervertebral Disc Degeneration in 2-Year-Old WT and GH receptor antagonist (GHA) Mice. (**a**) Safranin O/Fast Green staining of lumbar IVD comparing WT and GHA mice. IVD from WT mice display typical age-related degenerative changes, whereas GHA mice show relatively preserved disc structure with reduced severity of degeneration and suggesting a protective effect against age-associated IVD deterioration. Scale bar, 200 µm. (**b**) Bar graph showing the histological scores for different IVD regions. (**c**) Principal Component Analysis (PCA) illustrating clear separation between WT and GHA mice based on histopathological features. (**d**) Heatmap representing histopathological scores for different disc regions, including the NP, AF, and EP regions. Error bars represent mean ± SEM, statistical significance is denoted by **P < 0.01, ***P < 0.001 and ****P < 0.0001

We also examined intervertebral disc morphology in GHA mice, a line that expresses a growth hormone receptor antagonist, to determine whether suppression of GH signaling might confer protective effects. Remarkably, even at approximately 2 years of age, GHA mice exhibited minimal signs of disc degeneration. Safranin-O and Fast Green staining revealed preserved proteoglycan content and limited ossification in the IVD of GHA mice compared to age-matched WT controls (Fig. 3a; Figure S6). In contrast to the progressive deterioration seen in bGH mice, GHA mice maintained better structural integrity across all disc compartments.

Quantitative analysis confirmed that cumulative degeneration scores were significantly lower in GHA mice across all compartments (Fig. 3b). Principal component analysis (Fig. 3c) showed distinct clustering of GHA and WT controls. Individual histological scores further supported these observations, with GHA mice showing consistently lower degeneration scores across key parameters, including NP cellularity, NP matrix organization, AF cellularity, AF bulging, AF lamellar structure, AF clefts, and boundary cellularity (Fig. 3d). Collectively, these results support the notion that antagonizing GH signaling delays or protects against age-related intervertebral disc degeneration.

### Transcriptomic analysis reveals GH-induced molecular drivers of intervertebral disc degeneration with distinct sex-specific signatures

**Fig. 4:**
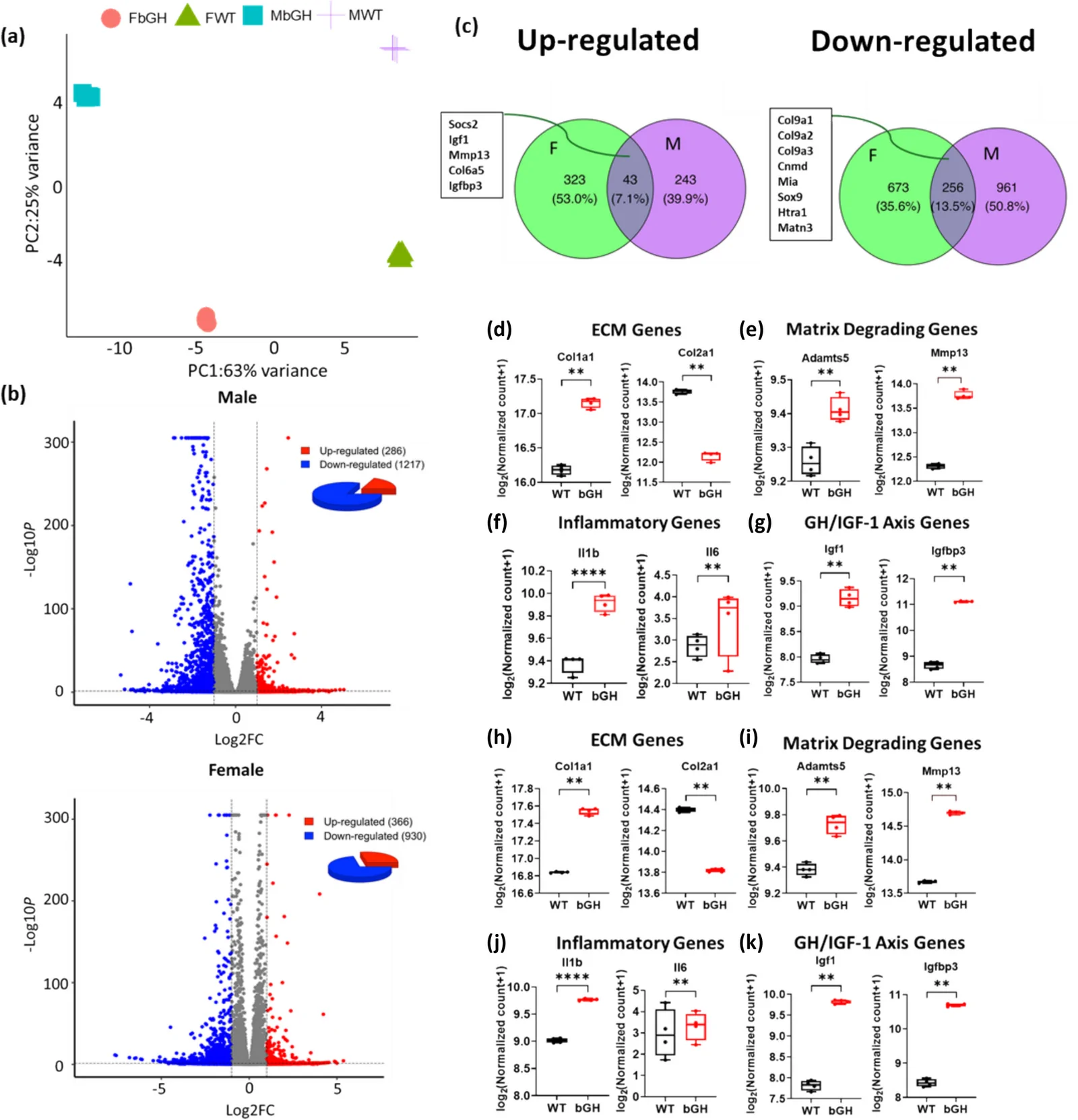
Comprehensive Gene Expression Analysis in WT and bGH Mice. (**a**) PCA Plot: Depicts the clustering of gene expression profiles by sex and genotype, demonstrating clear separation between male and female mice, as well as between WT and bGH groups. (**b**) Volcano plots display differentially expressed genes between WT and bGH mice in both sexes, with upregulated genes in red and downregulated genes in blue. The x-axis represents log₂ fold change, and the y-axis indicates –log₁₀ *p*-value. (**c**) Venn diagrams to illustrate the overlap of upregulated and downregulated genes in male (M) and female (F) mice, revealing sex-specific transcriptional responses to bGH overexpression. (**d**-**k**) Box plots illustrate expression levels of key genes related to cartilage matrix composition and inflammation—including *Col1a1, Col2a1, Adamts5, Mmp13, Il1b, Il6, Igf1*, and *Igfbp3*, between WT and bGH mice, highlighting significant group differences. Statistical significance is indicated as **P < 0.01 and ****P < 0.0001

To explore the potential mechanisms related to GH actions in IVD maintenance/degeneration, we conducted RNA Sequencing of the total IVD unit. RNA sequencing revealed widespread transcriptional differences between bGH and age-matched WT mice, with a PCA showing clear separation between bGH and WT mice in both sexes (Fig. 4a). The volcano plots indicated more genes crossing the significance threshold in males than in females, particularly those related to inflammatory responses and matrix remodeling. For example, catabolic genes such as Adamts4, Adamts5, Mmp9, and Mmp13 were significantly upregulated in bGH mice. However, the expression of Sox9 (Figure S7), a master transcription factor essential for chondrogenesis and extracellular matrix synthesis, showed reduced expression in bGH mice. Inflammatory markers genes (e.g., Il1b, Il6, and Tnf) were all significantly elevated in bGH mice, especially males (Fig. 4b). Venn diagrams visually depicted the distinct gene expression changes between males and females in response to bGH, with significant differences in the upregulated and downregulated genes between sexes (Fig. 4c). To further evaluate pathway-specific transcriptional alterations, we conducted a focused analysis of genes associated with the GH–IGF-1 signaling axis (Figure S7). In addition to the upregulation of Igf1 and Igfbp3 shown in Fig. 4, Ghr, Stat5a, Stat5b, Socs2, and Cish were examined. While Stat5a and Stat5b transcripts were modestly reduced, Socs2 and Cish were markedly upregulated, consistent with chronic GH pathway activation and feedback regulation. Examination of downstream IGF-1 receptor–mediated effectors revealed that Acan were significantly downregulated, whereas Timp1 expression increased and Bcl2 decreased in bGH discs compared with WT controls (Figure S7).

**Fig. 5:**
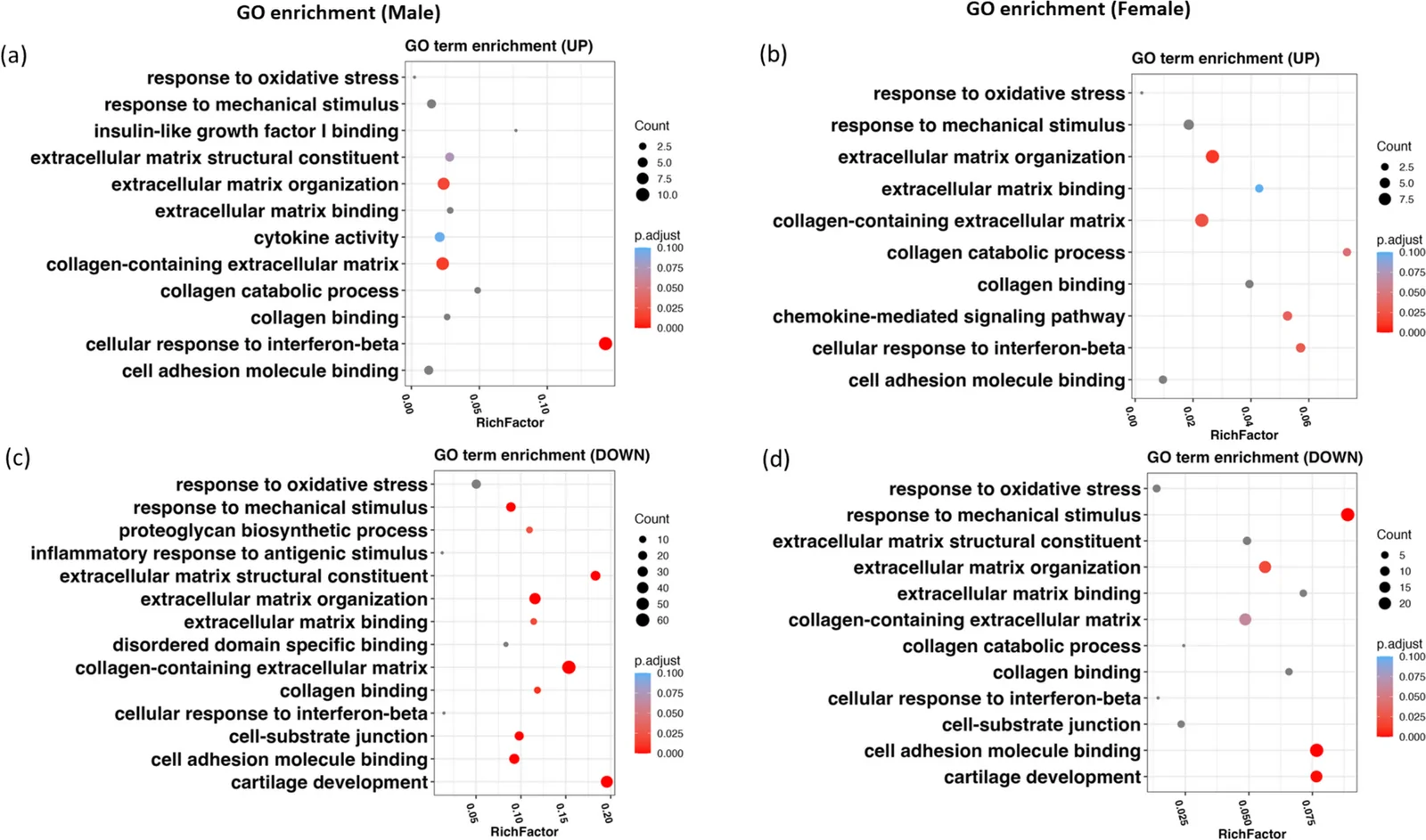
Differential Gene Ontology (GO) Term Enrichment comparing WT and bGH in Male and Female Mice. This figure shows a comparative Gene Ontology (GO) term enrichment analysis based on gene expression profiles in male and female mice. Panels (**a**) and (**b**) highlight GO terms significantly upregulated in males and females, respectively, with shared enrichment in processes such as "extracellular matrix organization" and "collagen catabolic process," suggesting common pathways with potentially distinct biological roles between sexes. Panels (**c**) and (**d**) present the downregulated GO terms in males and females, respectively, including pathways like "proteoglycan biosynthetic process" and "cartilage development," reflecting sex-specific molecular responses to physiological or experimental stimuli. Each dot represents an enriched GO term, plotted by its RichFactor—the ratio of genes involved in the term relative to the total number of background genes. Dot color indicates the adjusted *p*-value, with a blue-to-red gradient denoting increasing statistical significance

Gene Ontology (GO) term enrichment analysis was employed to describe the differential impact of genetic modifications on biological processes in male and female mice. Upregulated genes showed a significant enrichment in extracellular matrix organization and collagen catabolic process. These GO terms are crucial for maintaining structural integrity (Fig. 5a and c). Additionally, cellular response to interferon-beta and IGF1 binding were prominently upregulated in bGH mice. In contrast, the downregulated genes showed enrichment for proteoglycan biosynthetic process and cartilage development (Fig. 5b and d). The downregulation of these processes implies compromised tissue maintenance and repair capacity in the bGH mice.

**Fig. 6:**
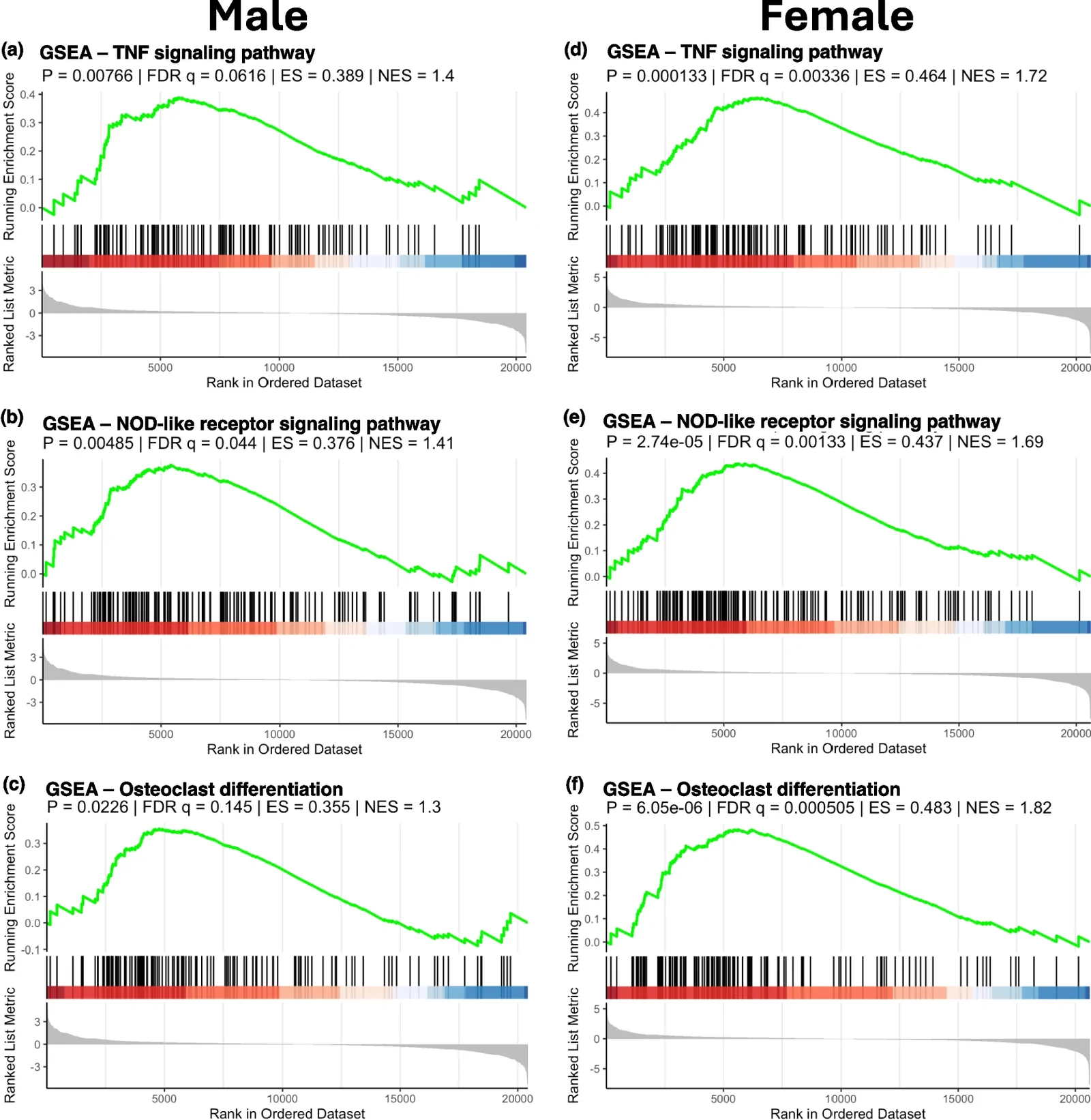
Comparative GSEA Analysis of Male and Female Mice. GSEA plots demonstrate significant enrichment of the TNF signaling pathway (**a**, **d**), NOD-like receptor signaling pathway (**b**, **e**), and osteoclast differentiation (**c**, **f**) in bGH versus WT mice. Males exhibit stronger enrichment across all pathways (higher NES and lower FDR) compared to females, indicating a more pronounced activation of pro-inflammatory and catabolic bone processes in male bGH mice

Gene Set Enrichment Analysis (GSEA) revealed robust enrichment of pro-inflammatory and catabolic signaling pathways in bGH intervertebral disc tissue, with distinct sex-specific patterns (Fig. 6, Supplemental Figure S3). These findings underscore the critical role of sex in modulating immune responses and disease progression. In male bGH mice, significant positive enrichment was observed in TNF signaling (NES = 1.72, FDR q = 0.00336), NOD-like receptor signaling (NES = 1.69, FDR q = 0.00133), and osteoclast differentiation pathways (NES = 1.82, FDR q = 0.00055), consistent with activation of cytokine networks, innate immune sensors, and matrix-degrading mechanisms. Supplemental Figure S3 further highlighted broad immune pathway activation in males, including NF-κB signaling (NES = 1.73, FDR q = 0.00285), IL-17 signaling (NES = 1.39), cytokine–cytokine receptor interaction (NES = 1.43), and Toll-like receptor signaling (NES = 1.38), pointing to a sex-biased amplification of the inflammatory network. Although female bGH mice also exhibited enrichment in these same inflammatory pathways, their normalized enrichment scores were lower (e.g., TNF signaling NES = 1.40, FDR q = 0.0616), suggesting a comparatively attenuated inflammatory phenotype. These results highlight the importance of including sex as a biological variable in mechanistic studies of disc degeneration. Both sexes showed negative enrichment in pathways associated with anabolic signaling and matrix homeostasis. However, this suppression was more pronounced in males, particularly for mTOR signaling (NES = –1.33), PI3K-Akt signaling (NES = –1.29), and calcium signaling (NES = –1.38), all of which are critical for disc cell viability and extracellular matrix maintenance. In females, these pathways were also modestly downregulated, albeit to a lesser extent. Collectively, these findings support a GH-induced, sex-dependent degenerative transcriptional signature in the IVD, marked by heightened immune activation and matrix degradation in males, and a comparatively moderated response in females.

The TNF and TGF-beta signaling pathways analysis assesses gender-specific differences in molecular responses (Figure S1 and Figure S2). The TNF signaling pathway analysis revealed a significant upregulation in the pro-apoptotic and inflammatory components of the pathway in males. Conversely, females exhibited a more moderated inflammatory and apoptotic response, with fewer significantly upregulated components. In addition, key inflammatory and apoptotic genes, including TNF, RIP1, CASP3, CASP7, IKKß, IL6, MMPs, and Ccls displayed marked upregulation in males but not females, suggesting a stronger inflammatory and apoptotic response to GH overproduction in males compared to females. The TGF-beta pathway analysis highlighted differences in cellular growth and differentiation signals between males and females. Males showed enhanced activity in pathways leading to cell cycle arrest and apoptosis through TGFßR1I and TGIF. In contrast, females demonstrated a higher regulation of genes involved in extracellular matrix synthesis and tissue repair (e.g., TGFßR1I/II and SMAD2/3). The TNF and TGF-beta signaling pathways highlight sex-specific-specific molecular mechanisms that may explain differences in disease progression and treatment responses between males and female.

## Discussions

Our study establishes a previously underappreciated role for growth hormone (GH) signaling in regulating intervertebral disc (IVD) homeostasis and degeneration, providing mechanistic insight into how GH excess accelerates disc deterioration and how GH antagonism confers protection, even during advanced aging. By combining histological, transcriptomic, and pathway-level analyses across multiple genetic mouse models, we demonstrate that GH action is a pivotal driver of degenerative changes within the disc and that its effects are both sex-dependent and transcriptionally distinct.

We first show that bGH transgenic mice, which mimic the elevated GH levels observed in patients with acromegaly, develop early and progressive disc degeneration. Even as early as three months of age, bGH mice exhibit histological signs of degeneration, namely, proteoglycan depletion, matrix disorganization, fibrosis, and increased NP cellularity. These changes were exacerbated in aged animals, particularly in males, where the degeneration extended across all IVD compartments. This phenotype parallels clinical observations in patients with acromegaly, who frequently suffer from disc-related pathologies such as disc herniation, spinal stenosis, and vertebral instability [14, 28, 50, 51]. However, while clinical studies often attribute these changes to increased mechanical load due to somatic overgrowth [52–54], our findings suggest that GH excess has a direct catabolic effect on the IVD itself, mediated by inflammatory and matrix-degrading pathways.

The apparent similarity in histologic scores between young and aged bGH mice likely reflects an early ceiling effect rather than the absence of progression. Because bGH mice display pronounced proteoglycan loss and architectural disruption by 3 months of age, subsequent aging primarily intensifies the qualitative severity of these lesions without substantially increasing the cumulative score. The scoring criteria primarily quantify structural parameters, which can appear similarly severe in both sexes once degeneration reaches an advanced stage. However, males exhibit a broader distribution of lesions, greater cellular hypertrophy, and more extensive fibrotic remodeling—features that may not proportionately increase the numerical score. This highlights an intrinsic limitation of cumulative scoring approaches in distinguishing subtle sex-dependent phenotypic variations.

Transcriptomic profiling provided strong support for this mechanism. We observed robust upregulation of key matrix metalloproteinases (e.g., Mmp9, Mmp13) and aggrecanases (Adamts4, Adamts5), along with marked increases in pro-inflammatory cytokines (Il1b, Il6, Tnf). Importantly, these molecular changes were more pronounced in male bGH mice, highlighting sex as a critical modifier of GH-driven pathology. This sex-specific response was also evident in gene set enrichment analyses, where males exhibited stronger activation of TNF, NOD-like receptor, and NF-κB signaling pathways, all of which are known to promote disc degeneration. Conversely, female bGH mice displayed a comparatively attenuated inflammatory profile and retained modest expression of ECM maintenance pathways. These results suggest a biological interaction between GH and sex hormones, potentially estrogen and its derivatives, in shaping disc vulnerability and maintenance.

Indeed, previous studies indicate that estrogen plays a crucial role in preserving disc integrity. For example, oophorectomy (surgical removal of ovaries) has been linked to a significantly heightened risk of degenerative spondylolisthesis, suggesting that estrogen withdrawal may accelerate disc degeneration [55]. The expression of estrogen receptors in intervertebral tissues has also been associated with the severity of degeneration in postmenopausal women [56]. MRI-based studies consistently show that postmenopausal women experience a faster progression of IVDD compared to age-matched men [57, 58]. Estrogen deficiency has been found to downregulate ECM components, such as collagen II and aggrecan, while simultaneously increasing the expression of degradative enzymes, including MMPs [59, 60]. In rat models, estrogen supplementation has been shown to reverse ovary removal-induced matrix degeneration and oxidative stress, suggesting a causal relationship [59, 60]. Notably, estrogen has been shown to antagonize GH action and suppress downstream IGF-1 production in clinical settings, as demonstrated in growth hormone-deficient women receiving oral estrogen therapy [61]. This mechanistic interplay may help to explain the sex-specific differences in GH-driven IVDD phenotypes observed in our study.

Additionally, we found that GH excess suppressed expression of genes involved in anabolic and repair processes, such as Sox9, an essential regulator in chondrogenic differentiation and ECM synthesis (Figure S7). Downregulation of proteoglycan biosynthesis and cartilage development-related GO terms further emphasizes the imbalance between catabolic and anabolic activities in the degenerated disc. Of note, both male and female bGH mice showed downregulation of PI3K-Akt and mTOR signaling pathways, both of which are central to cell survival and matrix production in disc cells [62, 63]. This disruption likely contributes to the loss of tissue integrity and regenerative potential in the bGH discs. Our focused analysis of the GH–IGF-1 signaling cascade revealed distinct transcriptional alterations across both upstream and downstream components of this pathway (Figure S7). Despite strong induction of Igf1 and Igfbp3 (Fig. 4) and elevated expression of feedback regulators (Socs2, Cish), we observed downregulation of Acan, along with reduced Bcl2 and increased Timp1. These findings suggest that chronic GH/IGF-1 activation elicits negative feedback and desensitization mechanisms that suppress anabolic matrix gene expression while enhancing stress and apoptotic responses.

In contrast, GHA mice—engineered to express a GH receptor antagonist—exhibited a strikingly preserved disc phenotype even at two years of age, a time point at which age-associated degeneration is commonly observed in WT mice. Histologically, the IVD in GHA mice maintained proteoglycan content, cellular organization, and boundary integrity across all compartments. Quantitatively, degeneration scores were consistently lower than WT controls, and PCA analysis confirmed a distinct histological profile. These results are particularly compelling given the advanced age of the animals and suggest that sustained inhibition of GH signaling is sufficient to protect against age-related IVDD. This finding aligns with previous work from our lab and others showing that disrupted GH actions extend both lifespan [35] and healthspan [64] in mice, but it now extends this protective effect specifically to tissues of the vertebral column, which had not been thoroughly investigated.

The divergent phenotypes of bGH and GHA mice also underscore the complexity of GH-IGF-1 signaling in IVD biology. Whereas IGF-1 has been shown to enhance matrix synthesis and cell viability in vitro and in injury models [18, 19, 65], previous published studies as well as our results highlight the context-dependent and dosage-sensitive nature of GH-IGF-1 action. IGF-1 promotes matrix synthesis in well-nourished disc regions, but it can also lead to increased cell mortality in nutrient-deprived areas [19]. IGF-1 can induce abnormal vascular ingrowth in human discs, a characteristic associated with painful, degenerated tissue [66]. Similarly, IGF-driven angiogenesis in degenerated discs and IGF-1 knockdown alleviated mechanical allodynia in a rat model of disc herniation, indicating its involvement in nociceptive signaling [67, 68]. Clinically, Koerner et al. reported that there is an elevated IGF-1 level in pain-associated regions in human samples of IVDD [69]. Furthermore, Zhang et al. discovered that while moderate doses of IGF-1 enhanced NP cell proliferation, higher concentrations of IGF-1 reduced cell viability [70]. Also, IGF-1 activates the PI3K/Akt pathway, leading to the expression of inflammatory cytokines in herniated discs [71, 72]. Our data suggests that chronic GH elevation may uncouple the beneficial effects of IGF-1 and instead drive pathological IVD remodeling via inflammation and ECM degradation. This is particularly relevant for clinical settings where GH or IGF-1 analogs are being considered for musculoskeletal disorders [73, 74]; our findings suggest that systemic GH modulation requires careful titration and consideration of sex-specific responses to avoid deleterious side effects.

Our study also contributes to a growing recognition of the importance of systemic and endocrine factors in disc degeneration, a field traditionally focused on biomechanics and local matrix biology [75]. The identification of GH as a systemic driver of disc pathology opens new avenues for understanding the interplay between endocrine aging and vertebral column health. Moreover, the sex-specific differences in GH-induced degeneration raise important questions about personalized approaches to treatment, especially in post-menopausal populations with altered and variable hormonal balance.

There are limitations to our study that merit discussion. First, while we assessed bulk gene expression across the IVD unit, future studies using single-cell RNA sequencing could more precisely define how GH affects specific cell types within the NP, AF, and EP. Second, although our models allow us to study long-term GH action, they do not disentangle the relative roles of GH versus IGF-1. Conditional knockout approaches targeting GHR or IGF1R in disc cells will be critical to delineate their individual contributions. Additionally, while we observed robust histological protection in GHA mice, functional outcomes such as pain behavior or biomechanical disc properties were not assessed and will be important for translating our findings into therapeutic strategies.

In summary, this study provides compelling evidence that GH signaling is a key modulator of intervertebral disc aging and degeneration. GH excess induces early and sex-dependent disc degeneration through inflammatory, apoptotic, and matrix-degrading pathways, whereas GH antagonism preserves disc architecture and delays degenerative changes. These findings not only enhance our understanding of the endocrine regulation of spine health but also raise the possibility that modulation of GH, either systemically or locally, could offer a novel strategies for preventing or treating chronic low back pain and IVDD.

## Supplementary Information

Below is the link to the electronic supplementary material.

Supplementary Material 1 (DOCX 10.8 MB)

## Data Availability

The datasets generated and analyzed during the current study are available from the corresponding author upon reasonable request. All relevant raw and processed data supporting the findings of this study can be shared for academic research purposes.
