# Supplementary material for: Intervertebral disc degeneration is accelerated by GH overexpression but attenuated by GH antagonism
Source: GeroScience. 2025 Dec 2;48(5):6817–32. doi: 10.1007/s11357-025-02022-9 (PMC13601400; doi:10.1007/s11357-025-02022-9)
Supplement: Supplementary file 1 [file 11357_2025_2022_MOESM1_ESM.docx]

**SUPPLEMENTARY FIGURES**

Continued….

**Figure S1: Comparative Analysis of TNF Signaling Pathway in Male and Female Mice**. This figure shows differential activation of the TNF signaling pathway in male and female mice. Expression levels are color-coded: blue indicates downregulation, red indicates upregulation, and white indicates no change. Key pathways and molecular interactions highlight the influence on apoptosis, necroptosis, and cell survival. The diagram emphasizes gender-specific differences in the TNF cascade, reflecting distinct regulatory mechanisms that affect inflammatory responses, apoptosis, and cellular homeostasis. Data are from a KEGG graph analysis rendered with Pathview to visualize pathway activity differences between sexes.

Continued….

**Figure S2: Comparative Analysis of TGF-Beta Signaling Pathway in Male and Female Mice.** This figure presents a comparative visualization of the TGF-beta signaling pathway in male and female mice, showing sex-specific responses to TGF-beta. The diagram color-codes expression levels: red for upregulation, blue for downregulation, and white for no change, based on gene expression data. Key nodes and interactions are highlighted, such as growth factor receptors, SMAD proteins, and transcription factors that regulate apoptosis, cell cycle, and differentiation. It also notes physiological processes that TGF-beta influences, including iron metabolism, osteoblast differentiation, and immune regulation. This representation highlights the complex regulatory mechanisms of TGF-beta and how these pathways differ by gender, affecting disease susceptibilities and therapy responses. The data is sourced from KEGG and visualized with Pathview software, mapping gene expression changes onto the signaling cascade, emphasizing distinct dynamics in male and female.

Continued….

**Figure S3:** Gene Set Enrichment Analysis (GSEA) of intervertebral disc transcriptomes from bGH and WT mice identifies sex-specific enrichment patterns in key signaling pathways. In male bGH mice (a–f), pathways associated with inflammation and tissue remodeling were modestly enriched or downregulated, including (a) Calcium signaling, (b) PI3K-Akt signaling, (c) mTOR signaling, (d) Rheumatoid arthritis, (e) Hedgehog signaling, and (f) ECM-receptor interaction. In contrast, female bGH mice (g–i) exhibited stronger upregulation of immune and cytokine pathways, including (g) IL-17 signaling, (h) NF-κB signaling, and (i) Cytokine–cytokine receptor interaction. Each panel shows the normalized enrichment score (NES), FDR q-value, and enrichment.

**Figure S4:** Higher-magnification view of Figure 1a showing lumbar intervertebral discs from 3-month-old WT and bGH mice. Safranin-O/Fast Green staining highlights fine structural details corresponding to the regions shown in Figure 1a. WT discs exhibit organized lamellar architecture and uniform proteoglycan distribution within the NP and AF. In contrast, bGH discs display disrupted NP structure, fibrotic remodeling, and endplate irregularities, with marked loss of proteoglycan staining and expansion of fibrotic matrix higher-resolution images provide detailed visualization of early degenerative changes associated with GH overexpression. Scale bar = 100 µm.

**Figure S5:** Higher-magnification view of Figure 2a showing lumbar intervertebral discs from 15-month-old WT and bGH mice. Safranin-O/Fast Green staining highlights detailed structural alterations corresponding to the regions shown in Figure 2a. WT discs display relatively preserved lamellar organization and proteoglycan distribution within the NP and AF, with mild age-related changes. In contrast, bGH discs show advanced degenerative remodeling, characterized by extensive proteoglycan depletion, dense fibrotic matrix deposition, irregular cartilage endplates, and disorganized NP-AF boundaries. These high-magnification images reveal severe architectural disruption and fibrosis associated with chronic GH overexpression. Scale bar = 100 µm.

**Figure S6:** Higher-magnification view of Figure 3a showing lumbar intervertebral discs from 2-year-old WT and GHA mice. Safranin-O/Fast Green staining highlights detailed morphology corresponding to the regions shown in Figure 3a. WT discs display age-associated proteoglycan loss and partial disorganization of the NP and AF. In contrast, GHA discs exhibit well-preserved NP structure, intact AF lamellae, and strong proteoglycan staining throughout the disc space, with minimal fibrotic remodeling. These high-magnification images demonstrate the marked preservation of extracellular-matrix integrity and disc architecture conferred by GH antagonism during advanced aging. Scale bar = 100 µm.

**Figure S7:** Focused analysis of GH–IGF-1 signaling–related genes in WT and bGH IVD. (a–j) males; (k–t) females. Expression of GH–STAT5 axis genes (Ghr, Stat5a, Stat5b, Socs2, and Cish) and IGF-1 downstream effectors (Igf1r, Sox9, Acan, Timp1, and Bcl2) in WT and bGH mice. Data represent log₂(normalized counts + 1) from RNA-seq. Stat5a/b are modestly downregulated, whereas Socs2 and Cish are strongly upregulated, indicating chronic GH pathway activation and feedback regulation. Downstream Sox9 and Acan are suppressed, while Timp1 increases and Bcl2 decreases, consistent with reduced anabolic signaling and elevated cellular stress in bGH discs.
